# Microevolution, speciation and macroevolution in rhizobia: Genomic mechanisms and selective patterns

**DOI:** 10.3389/fpls.2022.1026943

**Published:** 2022-10-25

**Authors:** Nikolay A. Provorov, Evgeny E. Andronov, Anastasiia K. Kimeklis, Olga P. Onishchuk, Anna A. Igolkina, Evgeny S. Karasev

**Affiliations:** ^1^ Laboratory of Microbiological Monitoring and Bioremediation of Soils, All-Russian Research Institute for Agricultural Microbiology, Pushkin, Russia; ^2^ Laboratory of Soil Biology and Biochemistry, V.V. Dokuchaev Soil Science Institute, Moscow, Russia; ^3^ Department of Applied Ecology, St. Petersburg State University, Saint-Petersburg, Russia; ^4^ Gregor Mendel Institute, Austrian Academy of Sciences, Vienna BioCenter, Vienna, Austria

**Keywords:** rhizobia, micro- and macro-evolution, speciation, natural selection, plant–microbe symbioses, evolutionary genomics, symbiotic N_2_ fixation, leguminous plants

## Abstract

Nodule bacteria (rhizobia), N_2_-fixing symbionts of leguminous plants, represent an excellent model to study the fundamental issues of evolutionary biology, including the tradeoff between microevolution, speciation, and macroevolution, which remains poorly understood for free-living organisms. Taxonomically, rhizobia are extremely diverse: they are represented by nearly a dozen families of α-proteobacteria (Rhizobiales) and by some β-proteobacteria. Their genomes are composed of core parts, including house-keeping genes (*hkg*), and of accessory parts, including symbiotically specialized (*sym*) genes. In multipartite genomes of evolutionary advanced fast-growing species (Rhizobiaceae), *sym* genes are clustered on extra-chromosomal replicons (megaplasmids, chromids), facilitating gene transfer in plant-associated microbial communities. In this review, we demonstrate that in rhizobia, microevolution and speciation involve different genomic and ecological mechanisms: the first one is based on the diversification of *sym* genes occurring under the impacts of host-induced natural selection (including its disruptive, frequency-dependent and group forms); the second one—on the diversification of *hkg*s under the impacts of unknown factors. By contrast, macroevolution represents the polyphyletic origin of super-species taxa, which are dependent on the transfer of *sym* genes from rhizobia to various soil-borne bacteria. Since the expression of newly acquired *sym* genes on foreign genomic backgrounds is usually restricted, conversion of resulted recombinants into the novel rhizobia species involves post-transfer genetic changes. They are presumably supported by host-induced selective processes resulting in the sequential derepression of *nod* genes responsible for nodulation and of *nif*/*fix* genes responsible for symbiotic N_2_ fixation.

## 1 Introduction

The trade-off between the diversification processes occurring at different phylogenetic levels—intra-species (microevolution), species, and super-species (macroevolution)—represents a puzzling issue in evolutionary biology. According to the Synthetic Theory of Evolution (STE) initially developed for the sexually reproducing organisms, speciation and macroevolution represent an extension of microevolution: local populations, biotypes, and subspecies formed under the impacts of natural, mostly individual (Darwinian) selection are presumably transformed into novel species, genera, and higher ranked taxa (Dobzhansky, 1951; Timofeeff-Ressovsky et al., 1977). However, Philiptschenko (1927) who coined the terms “microevolution” and “macroevolution,” as well as Koonin (2011) supposed that sufficiently different genetic mechanisms were responsible for these processes.

For eukaryotic organisms, tradeoff between micro- and macro-evolution remains obscure since adaptive impacts of macroevolutionary events, for example of macromutations in the master genes (e.g., in homeotic genes controlling the developmental regulation) are difficult to quantify in free-living plants or animals (Theißen, 2006). Symbiotic models are useful to address the adaptive impacts of developmental innovations resulting from the integration of hosts with microbial partners, which is best studied using the examples of root nodules in legumes (Bhattacharjee et al., 2015) and of light organs in squids (Peyer et al., 2014).

Broad opportunities to address the trade-off between different levels of evolution are provided by root nodule bacteria (rhizobia), N_2_-fixing symbionts of legumes (Fabaceae) from the Rosid I clade of dicot plants. These bacteria include a range of polyphyletically originated families and over a hundred species, mostly from α-proteobacteria (Rhizobiales) and some β-proteobacteria (e.g., *Paraburkholderia*) (Young, 1996; Zakhia and de Lajudie, 2001; Berrada and Fikri-Benbrahim, 2014; Wang et al., 2019). Several categories of symbiotically specialized (*sym*) genes of different origins were revealed in rhizobia including *nod* (synthesis of lipo-chito-oligosaccharidic Nod factors, NFs eliciting the root nodule development) (Gottfert, 1993; Debellé et al., 2001; Shamseldin, 2013), *nif* (synthesis of nitrogenase enzyme catalyzing N_2_ reduction to ammonium) and *fix* (energy supply of nitrogenase, *nif* gene regulation) (Shamseldin, 2013).

**Figure 1:**
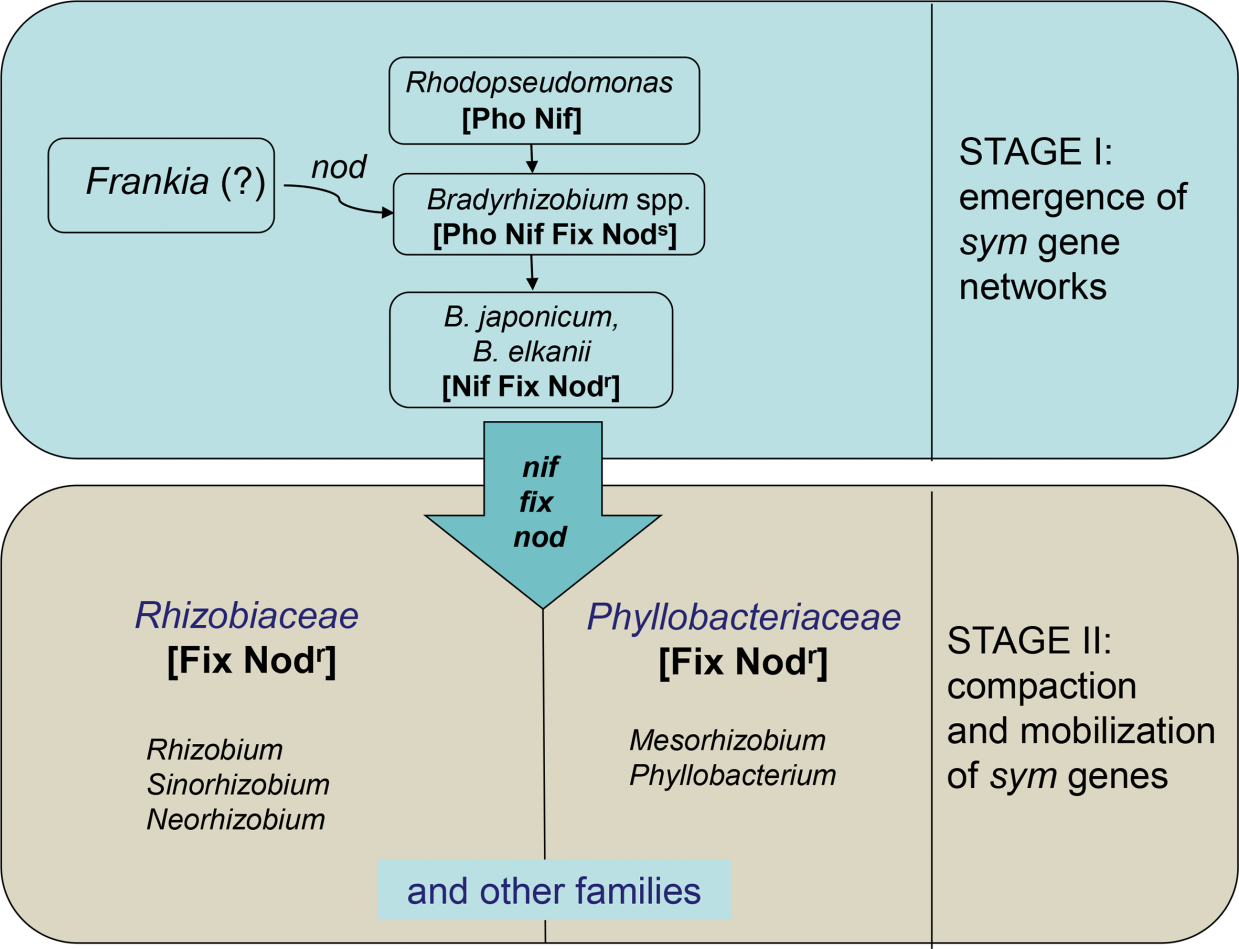
Rhizobia natural history: a two-stage scenario. Stage I: conversion of free-living diazotrophs (*Rhodopseudomonas*) into the primary rhizobia (*Bradyrhizobium*) *via* establishing the symbiotically specialized (*sym*) gene networks including genes for nodulation (*nod*) or N_2_ fixation (*nif*/*fix*). Stage II: emergence of secondary rhizobia *via* dissemination of *sym* genes among various families (e.g., Rhizobiaceae, Phyllobacteriaceae) of soil and plant-associated bacteria. Microbial phenotypes include: Pho, photosynthesis; Nif, free-living N_2_ fixation; Fix, symbiotic N_2_ fixation; Nod^s^, stem nodulation (crack entry); Nod^r^, root nodulation (root hair entry).

The extant rhizobia species may be classified into two categories emerged at different evolutionary stages (**Figure 1**): (1) primary (ancestral) species, emerged from free-living N_2_ fixers by genome rearrangements, resulting in formation of *sym* (*nod* + *nif*/*fix*) gene networks; (2) secondary (derived) species, originated *via* transfer of *sym* genes into various soil and plant-associated bacteria (Provorov and Andronov, 2016). The primary rhizobia represented by slow-growing *Bradyrhizobium* species close to *Rhodopseudomonas* presumably acquired the ability for *in planta* N_2_ fixation by allocating some photosynthesis-regulating genes into the nitrogenase-regulating *fix* network (Section 3.1).

The best studied secondary rhizobia are represented by fast-growing *Neorhizobium*, *Rhizobium*, and *Sinorhizobium* species (Rhizobiaceae) close to *Agrobacterium*, which is often addressed as the same genus as *Rhizobium* (Berrada and Fikri-Benbrahim, 2014; Ormeño-Orrillo et al., 2015; Wang et al., 2019). These symbiotically specialized bacteria are devoid of the ability to fix N_2_ *ex planta*, suggesting their origin *via sym* gene transfer from the preexisting rhizobia species. Transfer of large plasmids harboring *sym* genes as well as of chromosomally located *sym* islands may be responsible for the emergence of novel rhizobia genotypes and the distribution of *sym* genes in the microbial communities populating natural and agricultural ecosystems (Ding and Hynes, 2009). This transfer may stimulate recombination between IS copies, which are multiple in the symbiotically specialized genome regions providing the evolutionary important variation in rhizobia populations (Arashida et al., 2022).

An intriguing example of secondary rhizobia is represented by *Azorhizobium caulinodans* close to the free-living diazotroph *Xanthobacter autotrophicus* (Lee et al., 2008). This symbiont of tropical legume *Sesbania rostrata* combines the ability to fix N_2_ not only *in planta*, but also *ex planta* supporting the bacteria growth on N-free media (Boogerd et al., 1994). The genomic location of the *nod* cluster on a chromosomal island suggests the origin of *A. caulinodans* from a free-living N_2_ fixer *via* horizontal transfer of the *nod* gene cluster from a rhizobia species (Lee et al., 2008).

Rhizobia evolution involves two genomic strategies: “gain-and-loss of *sym* genes” and “compaction of *sym* gene clusters.” The gain of new *sym* genes from non-symbiotic networks occurred *via* horizontal gene transfer (HGT) and duplication–divergence (DD) mechanisms. In addition to *fix* genes, some *nod* genes have been recruited into symbiotic networks *via* the DD mechanism. For example, the *nod*D gene controlling expression of the *nod* regulon was obviously derived from the *lys*M-*ara*C family of transcriptional regulators by acquiring the ability to percept plant-released flavonoids (Hassan and Mathesius, 2012). However, “common” *nod* genes (*nod*ABC) which encode for the oligochitin part of the NF molecule were probably transferred from *Frankia*, the ancient N_2_ fixing symbiont of Rosid I plants, to some (presumably primary) rhizobia (Persson et al., 2015), which perhaps transmitted these genes to the other legume symbionts (*HGT-based emergency of rhizobia*).

The impact of gene loss on rhizobia evolution may be illustrated by *nif*V, which was revealed in phototrophic *Bradyrhizobium* genotypes. This gene encodes for the synthesis of homocitrate, a precursor for MoFe-cofactor of nitrogenase, which is usually supplied by plants, e.g., under the control of *FEN1* in *Lotus japonicus*. Inactivation of *FEN1* results in the loss of N_2_ fixation, which is restored after the introduction of *nif*V into *Lotus*-nodulating *Mesorhizobium loti* (Terpolilli et al., 2012). The *nif* gene losses occurred in many symbionts, e.g., primary rhizobia (*Bradyrhizobium*) retained 15–17 *nif* genes typical for free-living N_2_-fixers, while only 7–8 *nif* genes were found in the secondary rhizobia (*Rhizobium*, *Sinorhizobium*), which are not capable of *ex planta* N_2_ fixation (Pini et al., 2011). In later groups, a range of negative symbiosis regulators were revealed which inactivation by Tn5 insertions results in increased N_2_-fixing activity or nodulation competitiveness (Provorov et al., 2014; Onishchuk et al., 2017).

Compaction of *sym* gene arrangement was indicated in both primary and secondary rhizobia. The majority of *Bradyrhizobium* strains harbor *sym* genes in several chromosomal loci, while in some strains these genes are clustered on *Sym* plasmids (Okazaki et al., 2015). A compact arrangement was found in *Mesorhizobium* species in which *nod* and *nif* genes are clustered in chromosomal islands transferred in bacterial populations as conjugative transposons (Sullivan et al., 2002). In Rhizobiaceae species, an important factor for the high mobility of *sym* genes is represented by their clustering on the large *Sym* plasmids (pSyms) typical for *Rhizobium* and *Sinorhizobium* spp. (Poole et al., 2018). In *R. leguminosarum* bv. *viciae*, ancestral (A) strains isolated from nodules of *Vavilovia formosa*, a relict legume close to the common ancestor of the Fabeae tribe, possess a more scattered plasmid *sym* cluster (>90 kb) than the derived (D) strains isolated from *Pisum* and *Vicia* species (<60 kb) (Chirak et al., 2019). The A → D transitions involved a range of “gain-and-loss” events that presumably resulted in improved fitness in *R. leguminosarum* strains (**Table 1**).

**Table 1:** Differentiation of ancestral (A) and derived (D) groups of *Rhizobium leguminosarum* bv. *viciae* genotypes [from Chirak et al. (2019)].

Differentiating features	A group	D group	Possible impacts of A → D transition
Host affinities	*Vavilovia formosa* and primitive (“Afghan”) genotypes of *Pisum sativum*	Advanced (“European”) *P. sativum* genotypes, *Vicia* and *Lathurus* species	Adaptations towards new plant species emerged in the Fabeae tribe
Size of the extrachromosomal *sym* gene cluster, kb	>90	<60	Increased mobility of *sym* genes in the rhizobia populations
Location of *nod*T	Outside *nod* cluster	Inside *nod* cluster	Improved efficiency of Nod factor efflux
Presence of *nod*X	+	−	Narrowed host range
Presence of *fix*W	+	−	Deepened differentiation of N_2_-fixing bacteroids
Presence of the chromosomal *fix*NOPQ operon*	−	+	Improved fitness in microaerobic (soil, nodular) niches

*in addition to its plasmid-born copy involved in symbiosis. “+” – gene is present, “–” – gene is absent.

## 2 Microevolution and speciation: Divergence of rhizobia genomes

According to STE initially developed for sexually reproducing eukaryotic organisms, microevolution and speciation represent a continuum of biodivergence processes which are elicited by natural selection based on the differential ability of competing genotypes to produce fertile progeny (Dobzhansky, 1951; Timofeeff-Ressovsky et al., 1977). In contrast to microevolution, speciation results in a reproductive barrier: eukaryotic species are usually addressed as genetically closed systems in which recombination is restricted by intra-species hybridization, while the import of new genes *via* HGT is negligible (Katz, 2015).

For prokaryotes, these approaches are not valid since their reproduction is uncoupled from recombination, which occurs *via* parasexual processes resulting in HGT. Prokaryotic speciation does not mean a genetic barrier: gene exchange can be implemented between distant organisms, e.g., between bacteria and archaea (Fuchsman et al., 2017). The HGT-based gene flow provides the genomic cohesion of prokaryotic populations since the intensity of this flow is correlated to the relatedness of genotypes involved and may be used to suggest a biological species concept for prokaryotes which is at least partly analogous to the concept used for eukaryotes (Bobay, 2020). Importantly, prokaryotic species possess open genetic systems with pangenomes differentiated into the stable core parts which are transmitted vertically and the variable accessory parts for which HGT is intensive. The pangenome analysis may provide a new definition of prokaryotic species based on the identification of lineage-specific gene sets. While being similar to the classical biological definition based on allele flow, this definition does not rely on DNA similarity levels and does not require analysis of homologous recombination (Moldovan and Gelfand, 2018).

### 2.1 Divergence of core and accessory genomes

**Figure 2:**
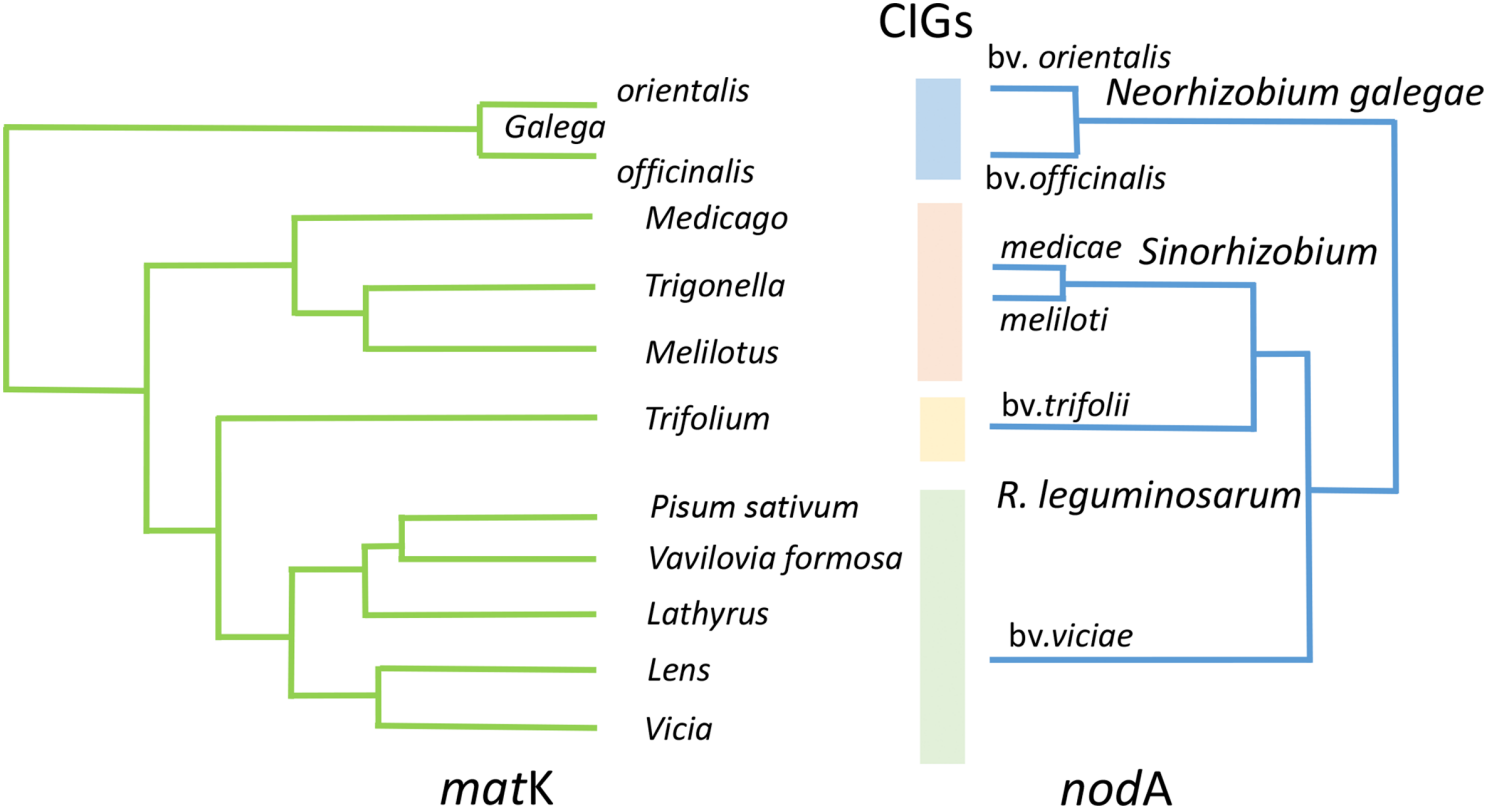
Phylogenetic congruence revealed in fast-growing rhizobia for *nod*A gene towards Galegoid legumes for *mat*K gene. Within the cross-inoculation groups (CIGs), a completely developed symbioses are formed which are usually characterized by active N_2_ fixation; between CIGs, the under-developed non-N_2_-fixing nodules may be formed rarely. Stylized phylogenetic trees were adapted using a graphic redactor from Azani et al. (2017) (*mat*K) and Haukka et al. (1998) (*nod*A) with the reduced number of nodes and the preserved initial topologies.

Suitable models to address the trade-off between intra-species diversification and speciation are represented by fast-growing rhizobia from the Rhizobiaceae family characterized by narrow host ranges towards the Galegoid legumes (**Figure 2**). We demonstrated (Kimeklis et al., 2018; Kimeklis et al., 2019) that in *Rhizobium leguminosarum*, the symbiotically contrasting biovars *viciae* (associated with the legume tribe Fabeae) and *trifolii* (associated with clovers from the Trifolieae tribe) are either not diverged for *hkg*s (p-distance analysis) or their divergence is sufficiently lower than for *sym* genes (group separation analysis).

**Figure 3:**
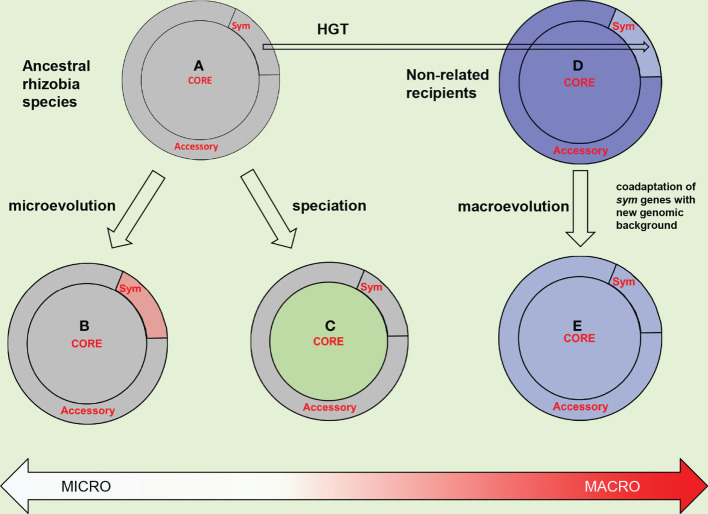
Trade-off between microevolution, speciation, and macroevolution in rhizobia. The proposed evolutionary paths are presented for an initial rhizobia species, which genome is differentiated into core and accessory parts, the later includes symbiotically specialized (*sym*) genes **(A)** Microevolution involves the divergence of *sym* genes (changed from gray for rosy) resulted in a host-specific (sym)biovar **(B)**. Speciation involves the divergence of core genes (changed for green) resulted in a cryptic (genomic) species **(C)**. Macroevolution is elicited by horizontal *sym* gene transfer (HGT) from rhizobia to genome of a non-related recipient (dark blue) **(D)**. Coadaptation of the acquired *sym* genes with the foreign genomic background (changed for light blue) **(E)** involve the host-induced selective pressures presented in **Figure 5**.

Importantly, within the host-specific biovars, variation for *hkg*s is much more pronounced than for *sym* genes suggesting that *hkg*-dependent speciation is not correlated to symbiotic diversification (Kimeklis et al., 2019). The complementary data were obtained using ANI technique suggesting that *hkg*s are diverged dramatically within a local *R. leguminosarum* population resulting in several genomic (cryptic) species which include bv. *viciae* and bv. *trifolii* strains (Kumar et al., 2015). Therefore, cryptic speciation represents a sympatric process which does not represent an extension of symbiotic diversification; they constitute two parallel pathways of divergent evolution (**Figure 3**).

The symbiosis-independent sympatric diversification of *hkg*s may be responsible for the emergence of novel species in different rhizobia groups. For example, a range of *Bradyrhizobium* species emerged in the Chinese center of soybean origin and retained similar host ranges. *B. japonicum* and *B. elkanii* strains produce N_2_-fixing nodules with *Glycine* spp. and other legumes from the symbiotically promiscuous Phaseoleae tribe, including *Cajanus*, *Phaseolus*, and *Vigna* (Lee et al., 2008).

Similarly, divergence of two sister *Sinorhizobium* (*Ensifer*) species, *S. meliloti* and *S. medicae* for the core genome markers occurred in spite of overlapping the geographic distribution and host ranges: these species differ for the ability to nodulate only some diploid medics, e.g. *Medicago polymorpha* (Rome et al., 1996; Bailly et al., 2006). Divergence for *hkg*s is also much stronger than for *sym* genes within *Neorhizobium galegae* host-specific biovars *orientalis* and *officinalis*, in which symbiotic N_2_ fixation is restricted to *Galega orientalis* and *G. officinalis*, respectively (Karasev et al., 2019).

Similar trends were revealed for plant pathogenic bacteria, in which the evolutionary dynamics of virulence genes differed greatly from the dynamics of *hkgs*. For example, in *Pseudomonas syringae*, differentiation into several dozens of host-specific pathovars determined by *vir* genes from accessory genomes differs greatly from phylogenetic groups determined by the core genome (Sarkar and Guttman, 2004; Słomnicka et al., 2015).

### 2.2 Ecological factors of rhizobia evolution

These factors may be classified into selective and stochastic ones operating in plant-associated rhizobia populations at the individual level (bacterial cells infecting the root hairs) or at the group level (cell groups maintained in individual plants or in different nodules formed on the same plant). The selective pressures may be positive or negative with respect to shifts in particular genotype frequencies resulting in their increase or decrease. With respect to gene structure, selective pressures may be differentiated into the purifying (stabilizing) and driving ones using the ratio of non-synonymous (dN) to synonymous (dS) substitutions: at dN/dS<1, selection is purifying, at dN/dS >1 it is driving while at dN/dS ≈ 1, the neutral, selection-independent evolution occurs (Kryazhimskiy and Plotkin, 2008).

#### 2.2.1 Host-specific diversification: Individual selection

Since in the majority of *sym* genes, expression is inducible under symbiotic conditions, the evolution of these genes is dependent on hosts. The resultant co-evolution of the partners is based on cross-regulation of genes encoding for host-symbiont recognition (e.g., bacterial *nod* genes for NF synthesis and the plant *NFR* genes for NF perception) (Broghammer et al., 2012) or for their metabolic integration (e.g., bacterial *nif/fix* genes for nitrogenase synthesis and plant *GS/GOGAT/AAT* genes for ammonium assimilation) (Betti et al., 2012). The ability of rhizobia genotypes for *in-planta* multiplication is based greatly on the production of NFs, eliciting the development of nodular niches for the propagation of bacteria. This selection may be highly efficient since the numbers of rhizobia cells released from the decayed nodules may exceed manifold their numbers in the soil (Yan et al., 2014).

Evolution of rhizobia populations under impacts of hosts may be presented as an interplay of Darwinian Selection (DaS) dependent on multiplication rates of co-inoculated genotypes, and of Frequency-dependent Selection (FdS) dependent also on the genotypic ratios in the inoculum (root-associated population) (Provorov and Vorobyov, 2006). Since soil rhizobia populations are highly polymorphic, severe competition occurs between virulent strains for occupation of nodular niches which are rich in C nutrients. Experimentally, a non-linear dynamics of rhizobia genotypic ratios was demonstrated for two-strain competition (Amarger and Lobreau, 1982):

***N*_1_: *N*_2_= *c*(** *I* **_1_**: ***I*_2_ )** ^***a***^ , where:

I_1_ and I_2_ are cell numbers of competing strains in the inoculum, N_1_ and N_2_—numbers in nodules formed by these strains; *c* is constant which may be either more or less than 1, demonstrating the strain competitiveness; *a* is constant which is uniformly less than 1 (usually, 0, 2< *a*< 0, 8).

From this dependence, it is evident that competition for nodulation results in negative FdS in favor of rare genotypes since N_1_:N_2_ > I_1_:I_2_ at I_1_< I_2_ (Provorov and Vorobyov, 2000; Onishchuk et al., 2017). The impact of FdS on mutualism evolution may be represented by an increased population diversity stimulated by the bacterial circulation in plant–soil systems. Deep amplicone sequencing of *nod*A gene libraries in *R. leguminosarum* populations composed of bv. *viciae* and bv. *trifolii* strains allowed us (Andronov et al., 2015) to address the host-induced selective pressures at two levels—genotypic (operational taxonomic units, OTUs identified at 97%–98% similarity) and haplotypic (individual sequences within the same OTU). We demonstrated that the number of OTUs identified for the *nod*A gene in nodules is lower than in soil since the avirulent and non-competitive genotypes are excluded from *in planta* multiplication. However, within symbiotically active OTUs, the diversity of haplotypes is usually increased, suggesting that the rare virulent strains are supported by FdS.

Additional selective pressures were induced during interaction of rhizobia populations with several hosts due to the preferential choice of some bacterial genotypes by legume plants (Andronov et al., 2015; Batstone et al., 2020). It was demonstrated that in *R. leguminosarum* the host preference pertains *nod* region located on *sym* plasmid, not the chromosomally located 16S–23S rRNA region suggesting the impacts of host plants on the microevolution of the symbionts, not on their speciation (Jorrin and Imperial, 2015). Due to the choice of the partners, co-vegetating legume hosts induce Disruptive Selection (DiS), resulting in a narrowed host range, which may be correlated with an improved N_2_ fixation rate (Provorov and Vorobyov, 2010).

**Figure 4:**
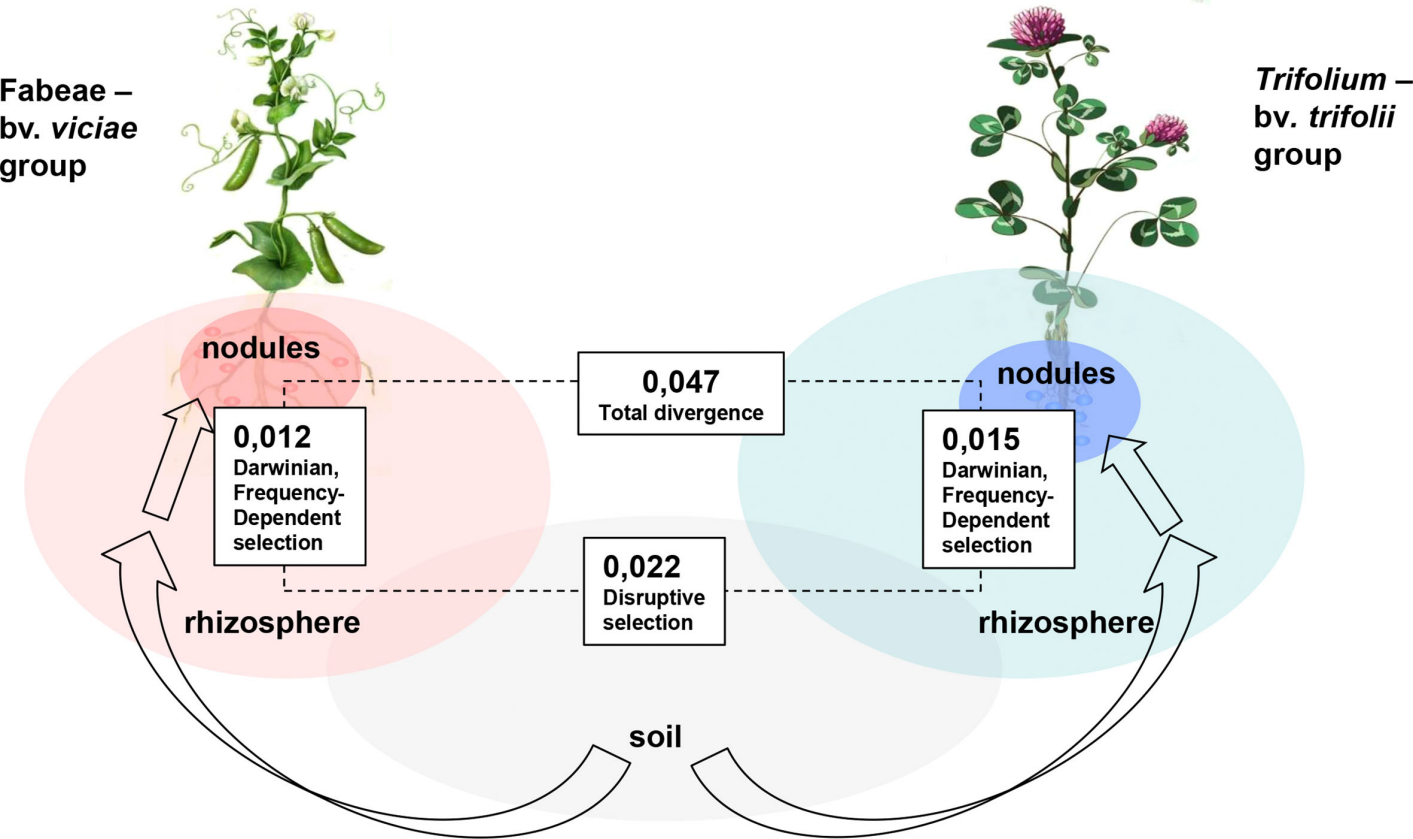
Diversification of *Rhizobium leguminosarum* population elicited by the natural selection pressures in symbiotic systems [adapted from Andronov et al. (2015)]. Arrows represent the bacteria migration into symbiotic niches, figures – p-distances between *nod*A in subpopulations formed in different niches under the impacts of host-induced selection: (i) Disruptive selection is elicited by rhizobia migration from soil to rhizospheres of hosts representing different cross-inoculation groups formed by the Fabeae tribe with bv. *viciae* and by *Trifolium* spp. with bv. *trifolii*; and (ii) Darwinian and Frequency-dependent selection are elicited by migration of bacteria from rhizospheres to nodules in each cross-inoculation group.

Importantly, host preference does not pertain to the *nif* genes, suggesting that hosts do not select symbionts directly for improved N_2_-fixing activity (Westhoek et al., 2017). However, host preference may result in an increased nodulation competitiveness of rhizobia strains which provide the indirect selective pressures for N_2_-fixing genotypes (Simonsen and Stinchcombe, 2014). Direct selection in favor of these genotypes may be due to sanctions against non-N_2_-fixing clones which occupy some nodules (Westhoek et al., 2017) or to stimulating propagation of active N_2_-fixers which occupy other nodules on the same root, collectively resulting in group-level (inter-deme, kin) selection for improved mutualism efficiency (*Section 2.2.2*). Analysis of the combined operation of FdS and DiS in the *R. leguminosarum* population composed of *viciae* and *trifolii* biovars may be done *via* comparison of subpopulations residing in rhizospheric and nodular niches of alternative hosts. It appeared that the p-distance between rhizosperic subpopulations of *Vicia/Lathyrus* and *Trifolium* is close to the sum of two p-distances between rhizospheric and nodular subpopulations calculated for each host (**Figure 4**), suggesting the nearly equal inputs of FdS and DiS in the symbiotic diversification of *R. leguminosarum* genotypes.

Host-induced selection in rhizobia populations results in the coevolution of the partners represented by shifts in population structure in a symbiotic organism under the impacts of population changes in its host (Janzen, 1980). These shifts result in the “evolutionary molding” (Igolkina et al., 2019), expressed as the congruent *nod* and *NFR* phylogenies and matching the diversity of NFs in rhizobial populations to the diversity of plant receptors. A pronounced congruence was also revealed when the phylogenies of galegoid legumes and their fast-growing symbionts (the Rhizobiaceae) were compared (**Figure 2**), but was not revealed for the higher ranked taxa of the partners (Provorov, 1998), probably due to multiple changes in host ranges induced by HGT in rhizobia populations (*Section 3.1*).

#### 2.2.2 Symbiotic N_2_ fixation: Group selection

According to the concept of symbiosis, coined by de Bary (1879), it represents a continuum of mutualistic and antagonistic interactions, which are similar in their mechanisms and are evolutionary interconnected. Being the founder of phytopathology, de Bary assigned a leading role in this continuum to parasitism, which can be sometimes reorganized into mutualism under the impacts of selective pressures favoring reciprocally beneficial cooperation (Dobzhansky, 1951). Lewis (1974) suggested that these reorganizations were related to nutritional strategies in symbiotic microbes that evolved from necrotrophic to biotrophic and symbiotrophic ones.

Simulation of population dynamics in symbiotic organisms suggests that individual selection explains readily the evolution of antagonism but not of mutualism (Wyatt et al., 2013), which is sometimes addressed as a side-effect of individual adaptations (Smith, 1989). Specifically, DaS models fail to explain the rhizobia evolution for irreversible differentiation into N_2_-fixing bacteroids, which may be addressed as temporary organelles of plant cells (Coba de la Peña et al., 2017), since the bacteroid operation is considered “altruistic” towards the legume hosts (Provorov, 2021).

In order to reconcile the theory of natural selection with the evolution of beneficial cooperation, one can suggest that the selective pressures favoring mutualism are implemented within the endosymbiotic microbial populations under host impacts. In nodular symbiosis, these pressures should be related to the positive feedbacks of partners: the intra-nodular rhizobia groups (which are often represented by clonal progenies of individual cells) if fixing N_2_ actively, obtain a preferential C supply from hosts which support not only the nitrogenase activity but also the *in planta* propagation of N_2_-fixing bacterial genotypes (Udvardi and Kahn, 1992). The population structures of micro-symbionts favorable for this selection result from inoculation mechanisms evolved from the crack entry that leads to the mixed rhizobial infection, towards the root hair entry by individual cells or micro-colonies which favors the “clonal endophytes” (Brewin, 1998; Sprent, 2001; Brewin, 2004).

Experimental evidence for preferential C supply of Fix^+^ soybean nodules, which may be either provided with or devoid of N_2_ (Denison and Kiers, 2004), suggests a positive *in planta* selection in favor of the clonally propagated Fix^+^ genotypes. This propagation may be also supported by negative selection against Nod^+^Fix^−^ genotypes due to host-induced “sanctions” based on nutrient restrictions or on defense reactions (Denison, 2000). Collectively, these mechanisms may promote group (inter-deme) selection in favor of N_2_-fixing genotypes within the nodular rhizobia populations.

## 3 Macroevolution: Divergence based on gene transfer

For eukaryotic organisms, the emergence of superspecies taxa (macroevolution) is usually considered as an extension of microevolution and speciation as adaptive processes dependent on natural selection. Rhizobia provide the opportunity to address the validity of this approach for prokaryotes by dissecting the genomic and ecological mechanisms of their macroevolution. It involves the emergence of symbiotic N_2_-fixers from free-living bacteria acquiring the *sym* gene systems *via* two processes: (i) genomic rearrangements in the ancestral N_2_ fixers (*Divergence of core and accessory genomes*); and (ii) transfer of *sym* genes from rhizobia to diverse soil-borne bacteria (**Figure 1**).

### 3.1 HGT-based emergency of rhizobia

As we indicated previously, emergence of primary rhizobia (*Bradyrhizobium*) from free-living phototrophic N_2_-fixers (*Rhodopseudomonas*) involved: (i) allocation of some photosynthesis-controlling genes into nitrogenase-controlling network in free-living bacteria which resulted in the root-associated genotypes to be used for rice crop fertilization (Maeda, 2022) and may be further evolved into phototrophic stem-nodulating rhizobia (see below); (ii) acquisition of *nod* genes *via* HGT resulted in root-nodulating genotypes (Ding and Hynes, 2009; Arashida et al., 2022). The hypothesis of the direct filiation of *Rhodopsedomonas* into primary rhizobia (*Bradyrhizobium*) is supported by the transitional forms represented by phototrophic bradyrhizobial strains devoid of *nod* genes (Mornico et al., 2012). A possibility to consider the legume-nodulating β-proteobacteria (e.g., *Paraburkholderia*) as the primary rhizobia may be discussed since some representatives of this bacterial group were identified as endosymbionts of Glomerymycotan fungi (Pawlowska et al., 2018), the ancient symbionts of land plants forming arbuscular mycorrhiza which perhaps donated some of their bacterial symbionts to the plant hosts (Provorov and Shtark, 2014).

The first process involves the conversion of some photosynthesis-controlling genes into nitrogenase-controlling ones (e.g., *cc_3_* NOPQ into *fix*NOPQ) (Rey and Harwood, 2010). This reorganization resulted in photosynthetically active *Bradyrhizobium* genotypes nodulating the stems in some tropical legumes (e.g., *Aeschynomene*) *via* the crack entry without using NFs typical for majority of rhizobia (Sprent, 2001). The NF synthesis has been acquired by bradyrhizobial species (e.g., *B. japonicum*, *B. elkanii*) nodulating the legumes *via* root hair infection. In these bacteria, phototrophy was functionally substituted by the ability to use plant photosynthesis products. The resulted bradyrhizobia often retain the *ex planta nif* gene expression, but they are usually not capable of diazotrophic growth due to low free-living nitrogenase activity (Wongdee et al., 2018). The emergence and evolution of primary rhizobia involved the enlargement of individual genomes and of pangenomes, which was based on the extension of their accessory parts (**Table 2**).

**Table 2:** Genomic features of free-living (*Rhodopseudomonas*) and symbiotic (*Bradyrhizobium*) members of the Bradyrhizobiaceae.

Bacteria	Average numbers of genes in:		% of accessory genes in:		References
	Individual genomes	Pangenomes	Individual genomes	Pangenomes	
*Rhodopseudomonas*	5,408	8,000	22.5	52.7	Oda et al. (2008)
*Bradyrhizobium* (phototrophic)*	7,110	12,040	33.6	60.2	Mornico et al. (2012)
*Bradyrhizobium* (heterotrophic)**	9,821	>35,000	72.0	>92.0	Tian et al. (2012)

*strains devoid of *nod* genes and nodulating the stems of *Aeschynomene* not using Nod factors.

**over 15 species from two groups represented by *B. japonicum* and *B. elkanii* nodulate the roots in diverse legumes (mostly from the Phaseoleae tribe) using Nod factors encoded by *nod* genes.

The *nod* genes encoding for the NF synthesis may be acquired by rhizobia from actinobacteria *Frankia*, which are ancient N_2_-fixing symbionts of Rosid I dicots. Specifically, some *Frankia* strains possess common *nod*ABC genes which are activated during host (*Datisca glomerata*) nodulation and are functionally interchangeable with the rhizobial *nod* genes (Persson et al., 2015). Importantly, NodA-like acyl transferases are found in diverse actinobacteria, while in α-proteobacteria these enzymes are restricted to rhizobia. When acquired *nod*ABC, ancestral rhizobia possibly substituted *Frankia* in the endosymbiotic niches due to a more rapid multiplication of unicellular α-proteobacteria as compared to multicellular actinobacteria (Provorov and Vorobyov, 2010).

Subsequent evolution of the Rhizobiales was presumably due to HGT-based polyphyletic emergence of multiple rhizobia taxa induced by co-migration of symbionts with their hosts into the novel areas wherein *sym* genes were donated by introduced bacteria to the local ones (**Table 3**). This evolution is well documented for symbionts of polebean (*Phaseolus vulgaris*), which in the Central- and Southern-American centers of origin is associated with *R. tropici* and *R. etli* (Eardly et al., 1995; Bernal and Graham, 2001; Aguilar et al., 2004). In Europe where the polebean was introduced in Columbian times, a broad spectrum of new symbionts emerged, including *R. gallicum*, *R. giardinii*, and *R. leguminosarum* bv. *phaseoli* (Amarger et al., 1994; Laguerre et al., 2001; Martínez-Romero, 2003). These rhizobia harbor a range of *nod* markers common to *R. tropici* and *R. etli*, suggesting the similarities of NFs synthesized by ancestral and derived polebean symbionts (Laguerre et al., 2001).

**Table 3:** Emergence of novel symbionts of legumes by transfer of *sym* genes to local bacteria from rhizobia migrated to the novel ecological areas with the help of plant vectors.

Migrated rhizobia	Transferred genomic elements	Newly emerged symbionts	Plant vectors	Direction of migration (its tentative age in years is given in parenthesis)	References
*Mesorhizobium loti*	Chromosomal islands	*Mesorhizobium* spp.	*Lotus corniculatus*	Europe → New Zeeland (7)	Sullivan et al. (1995; 2002)
			*Biserrula pelecinus*	Mediterranean area → Australia (12)	Nandasena et al. (2006)
		*Mesorhizobium* spp.*, Rhizobium* spp.	*Amorpha fruticosa*	North America → China (50)	Wang et al. (1999)
			*Robinia pseudoacacia*	North America → Europe (300)	Ulrich and Zaspel (2000)
*Rhizobium tropici, R. etli*	*Sym* plasmids	*R. leguminosarum* bv. *phaseoli, R. gallicum, R. giardinii*	*Phaseolus vulgaris*	South and Central America → Europe (500)	Laguerre et al. (1993); Bernal and Graham (2001); Laguerre et al. (2001); Brom et al. (2002)

A rapid generation of novel symbionts *via* recombination of introduced rhizobia with local bacteria was demonstrated for *Mesorhizobium* spp. harboring *sym* genes in chromosomal islands, which may be transmitted to local bacteria as the conjugative transposons *via* type 4 secretion systems (Sullivan et al., 2002). This transmission was demonstrated for the trefoil (*Lotus corniculatus*) rhizobia introduced from Europe to New Zealand, wherein the novel symbiont populations were established in a few years (Sullivan et al., 1995; Sullivan et al., 2002). Similar processes accompanied the co-introduction of *Mesorhizobium* spp. with *Robinia pseudoacacia* from North America to Europe, *Amorpha fruticosa* from North America to China, and *Biserrula pelecinus* from the Mediterranean area to Australia (**Table 3**).

### 3.2 *sym* gene activation

**Figure 5:**
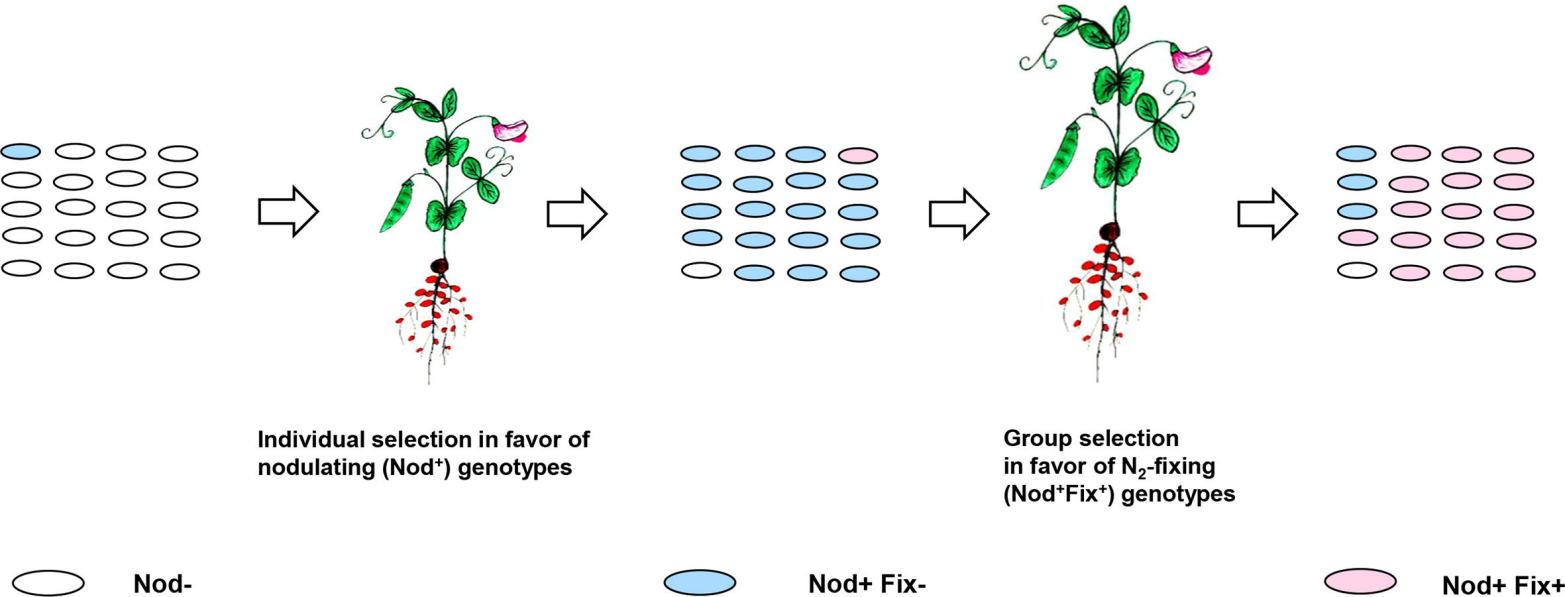
Derepression of the newly acquired *sym* genes in recombinant genotypes supported by host-induced selective pressures. Individual selection (*Section 2.2.1*) in a population of non-nodulating (Nod^−^) recombinants generated by transfer of *sym* genes results in an increased frequency of Nod^+^Fix^−^ genotypes generated *via nod* gene derepression. Group selection (introduced in *Section 2.2.2*) occurs in favor of N_2_-fixing (Nod^+^Fix^+^) genotypes (with the derepressed *nif/fix* genes) propagated actively in N_2_-fixing nodules due to their preferential carbon supply.

Obviously, the HGT-based emergence of novel rhizobia species may be restricted by the poor expression of transferred *sym* genes in the foreign genomic background. For example, after p*Sym* transfer from *Rhizobium* and *Sinorhizobium* species to closely related agrobacteria, low virulent, non-N_2_-fixing recombinants usually emerge. Few reports are available on the emergency of N_2_-fixing recombinants *via* transfer of p*Sym*s into agrobacteria from the broad-host-range rhizobia, e.g., from *R. tropici* (Rogel et al., 2001). The recombinant genotypes resulting from *sym* gene transfer from rhizobia to distant bacteria are mostly non-virulent (reviewed in Provorov and Vorobyov (2010)). Therefore, additional genomic changes accompanied by host-induced selective pressures were required to support the symbiotically active strains in which the newly acquired *sym* genes were functional (**Figure 5**).

Analysis of host-induced selective processes involved in rhizobia evolution (*Section 2.2*) suggests that conversion of non-virulent recombinants into active nodulators may be dependent on individual selection in favor of virulent genotypes with derepressed *nod* genes, providing the ability of bacteria to actively propagate *in planta*. Positive selection for *nod* gene derepression (Nod^+^ phenotype) may be highly effective if the rare virulent rhizobia cells are picked by plants from mixtures with numerous non-virulent bacteria (Clúa et al., 2018).

However, Nod^+^ strains supported by individual selection may be represented mostly by non-N_2_-fixing cheaters, which require additional genetic changes to be transformed into N_2_-fixing (Fix^+^) mutualists. The relevant selective pressures possibly operate *in planta* at the level of intra-nodular clones: Fix^+^ genotypes may be supported by group (inter-deme) selection (Provorov, 2021). It favors strains with *nif/fix* gene derepression propagated due to the active supply of bacteria with the plant photosynthesis products based on their exchange for N_2_ fixation products (**Figure 5**).

## 4 Towards a multilevel classification of evolutionary processes (conclusion)

In this paper, we use nodule bacteria as a model to study the interplay of microevolutionary, speciation, and macroevolutionary processes. For eukaryotic organisms, their trade-off is conventionally considered as hierarchic and reductionist: speciation and macroevolution are addressed as an extension of microevolution (Dobzhansky, 1951; Koonin, 2009). According to STE, these processes are driven by individual selection, which supports genotypes with high fitness measured as the production of fertile progeny (Timofeeff-Ressovsky et al., 1977; Koonin, 2011). However, different genetic mechanisms were proposed for micro- and macro-evolution by J. Philiptschenko (1927), who suggested that in eukaryotic organisms these processes are associated with reorganizations of nuclear and cytoplasmic genes, respectively.

We suggest that diverse evolutionary strategies are implemented in rhizobia at different phylogenetic levels and result in the gene, genomic, and phylogenomic reorganizations responsible for the rhizobia microevolution, speciation, and macroevolution (**Table 4**). Divergent evolution based on genomic reorganizations and driven by disruptive selection occurs mostly at the species and subspecies levels, while at the superspecies level, HGT-based (reticular) evolution is implemented. These reorganizations are elicited by host-induced and environmentally-dependent selective factors, which include symbiosis-specific forms of natural selection operating at the individual and group levels. The impact of these factors is evident also for the macroevolutionary processes responsible for conversion of HGT-born recombinants into novel rhizobia species (**Figure 5**). Importantly, in rhizobia, the symbiotically specific natural selection pressures induce modifications of *sym* gene clusters, which represent the most active evolutionary part of the dispensable (accessory) gene pool driving evolution of the whole bacterial genomes.

**Table 4:** Multilevel classification of evolutionary processes in rhizobia based on reorganizations of symbiotically specialized (*sym*) and of housekeeping genes (*hkg*).

Genetic impacts registered at:	Levels of divergence		
	Sub-species(microevolution)	Species	Super-species (macroevolution)
Gene level (selective factors involved)	Divergence of *sym* genes (disruptive selection elicited by plant hosts possessing different symbiotic affinities)	Divergence of *hkg*s (selective pressures presumably induced by soil environment)	Horizontal transfer of *sym* genes to diverse recipients (host-induced selection for *sym* gene de-repression*)
Genomic level (molecular mechanisms involved)	Reformatting of *sym* gene clusters (genome rearrangements, horizontal gene transfer)	Modifications of genome architecture (its internal rearrangements and acquisition of new genes; Section 3.1)	Emergence of *sym* gene networks, their allocation to special replicons or islands (increased gene mobility)
Phylogenomic level: new taxa emerged	(sym)biovars emerged within an ancestral species	Genomic (cryptic) species diverged from an ancestral one	Super-species taxa capable to occupy the symbiotic niches

*specified in **Figure 5**.

Rhizobia provide broad opportunities to study the coevolution of partners in mutualistic symbioses. According to the definition coined by Janzen (1980), coevolution involves the inter-dependent changes in population structures of tightly interacting species. Up to now, co-evolutionary processes have been studied mostly in antagonistic symbioses wherein “gene-for-gene” interactions between parasites and hosts are implemented (Jones and Dangl, 2006). These interactions are controlled by individual selection operating in the Darwinian and frequency-dependent forms; it usually results in the coordinated oscillations in frequencies of virulence and resistance genes encoding the specificity of interactions between the partners (Luijckx et al., 2013).

A range of similar molecular and ecological mechanisms involved in the coevolution of the partners were revealed in mutualistic symbioses. In the legume–rhizobia system, co-evolution may be represented as “evolutionary molding,” matching the rhizobia population diversity for *nod* genes encoding for NF synthesis to the host diversity for NF-specific receptors (Igolkina et al., 2019). This co-evolution results in the narrowing specificity of the interactions of the partners, which may be correlated to an increased benefit of their co-operation (Provorov and Vorobyov, 2010).

For future research, it would be interesting to address the legume-rhizobia coevolution for the components of cooperative metabolic pathways, linking the bacterial *nif/fix* genes for nitrogenase synthesis with the plant *GS/GOGAT/AAT* genes for assimilation of fixed nitrogen or the bacterial genes encoding for catabolism of C compounds with the plant genes responsible for providing these compounds for bacteroids (Udvardi and Poole, 2013). From a perspective, application of the rhizobia–legume model will allow us to represent the coevolution of the partners for signaling and metabolic interactions as the natural history of an integral holobiont possessing a hologenome encoding for the cooperative adaptations of tightly integrated organisms to adverse environments (Zilber-Rosenberg and Rosenberg, 2008). Specifically, legume genes for hosting rhizobia may be addressed as homeotic (master) genes since they allow plants to switch on the novel developmental program providing the adaptively valuable symbiotrophic N nutrition (Bhattacharjee et al., 2015).

Speaking generally, symbiotic models provide clear examples of punctuated evolution (Gould and Eldredge, 1993), since the hosting of symbiotic microbes represents the rapid evolutionary bursts in contrast to gradual evolution suggested by the conventional models of natural selection (Gould, 1989). In symbiotic systems, hypothetical “hopeful monsters” may be replaced by actual “successful cooperators” in which the increased fitness is due to functional and structural innovations resulting from the integration of hosts with symbiotic microbes into the holobiont/hologenome units (Theis et al., 2016). In these units, the non-friendly, competitive, and antagonistic interactions of partners may be reorganized into beneficial symbioses, providing the broad prospects for adaptive and progressive coevolution of partners (Douglas, 2014).

## Author contributions

NP—project conceptualization, manuscript preparation, and funding acquisition. EA—project conceptualization and manuscript editing. AK—manuscript preparation. OO—collection of literature and manuscript proofreading. AI—project conceptualization and data processing. EK—project conceptualization, data processing. All authors contributed to the article and approved the submitted version.

## Funding

This study is supported by the Russian Science Foundation, grant 19-16-00081P.

## Acknowledgments

Authors are grateful for the contribution of the Russian Collection of Agricultural Microorganisms (RCAM, WDCM 966 supervised by Dr. V. Safronova) and the Centre for Genomic Technologies, Proteomics and Cell Biology in ARRIAM.

## Conflict of interest

The authors declare that the research was conducted in the absence of any commercial or financial relationships that could be construed as a potential conflict of interest.

## Publisher’s note

All claims expressed in this article are solely those of the authors and do not necessarily represent those of their affiliated organizations, or those of the publisher, the editors and the reviewers. Any product that may be evaluated in this article, or claim that may be made by its manufacturer, is not guaranteed or endorsed by the publisher.
